# Endoplasmic reticulum stress and exosomes secretion in the pathogenesis of inflammatory bowel disease: a concise summary of research findings

**DOI:** 10.3389/fphar.2025.1702825

**Published:** 2025-11-26

**Authors:** Ashiq Shibili P, Antara Banerjee, Soham Chakraborty, Suresh Babu Kondaveeti, Andrea Porzionato, Silvia Barbon, Surajit Pathak

**Affiliations:** 1 Medical Biotechnology Lab, Faculty of Allied Health Sciences, Chettinad Academy of Research and Education (CARE), Chettinad Hospital and Research Institute (CHRI), Chennai, India; 2 Department of Biochemistry, Symbiosis Medical College for Women, Symbiosis International (Deemed University), Pune, India; 3 Section of Human Anatomy, Department of Neuroscience, University of Padova, Padova, Italy

**Keywords:** endoplasmic reticulum stress, exosomes, immune dysregulation, inflammatory bowel disease, intercellular communication, unfolded protein response

## Abstract

Cellular stress responses and intercellular communication play a crucial role in the pathogenesis of Inflammatory bowel disease (IBD). Among these, endoplasmic reticulum (ER) stress and exosome-mediated signaling have emerged as interconnected drivers of chronic intestinal inflammation. Persistent ER stress, primarily through unfolded protein response pathways involving PERK, IRE1, and ATF6, disrupts epithelial barrier integrity, alters immune cell function, and promotes pro-inflammatory gene expression. ER stress not only affects intracellular homeostasis but also modulates intercellular communication through the secretion of exosomes, which carry proteins, lipids, and nucleic acids. This bidirectional relationship ensures that stress-altered exosomes can amplify ER stress and inflammatory signals in neighboring cells, sustaining intestinal inflammation. For this review, relevant research and review articles were retrieved from established search engines and databases, including PubMed, Google Scholar, and ScienceDirect, using key terms such as “endoplasmic reticulum stress,” “exosome secretion,” “exosome cargo,” “inflammatory bowel disease,” “intestinal inflammation,” and “intercellular communication.” The literature search primarily focused on studies published in the last 5 years, prioritizing clinical and preclinical studies (*in vivo* and *in vitro* models). Published literature addressing ER stress, exosome biology, and their interconnection in IBD were included, whereas studies lacking relevance or study quality were excluded. Recent findings highlight a dynamic interconnection between ER stress and exosomes, where ER stress modulates exosome biogenesis, secretion, and cargo composition. In contrast, stress-altered exosomes amplify ER stress signals and inflammatory mediators in neighboring cells. This review aims to summarize the current evidences on the interconnection of ER stress and exosomes in modulating the intestinal microenvironment, driving inflammation, and contributing to epithelial and immune dysregulation in IBD. This review also highlights experimental insights, existing challenges, and therapeutic prospects for targeting the ER stress–exosome axis to restore mucosal homeostasis in IBD management.

## 1 Introduction

Inflammatory Bowel Disease (IBD) is a chronic and relapsing inflammatory condition of the gastrointestinal tract, primarily encompassing two major clinical entities: Crohn’s disease (CD) and Ulcerative colitis (UC) (Hirten et al., 2024). While UC is limited to the colon and rectum, CD may affect any region of the gastrointestinal tract, often resulting in complications such as bowel strictures, fistulas, malnutrition, anemia, and hepatic disorders (Ber et al., 2021). Globally, IBD has emerged as a significant public health concern, with incidence rates that were once confined to high-income Western countries now increasing rapidly in newly industrialized regions such as East Asia and Latin America (Lin et al., 2024; Park et al., 2023). This epidemiological shift is largely attributed to industrialization, urbanization, dietary transitions, and other environmental factors interacting with genetic susceptibility (Shan et al., 2022). According to Global Burden of Disease (GBD) analyses, the age-standardized incidence of IBD showed a modest increase, rising from 4.22 per 100,000 individuals in 1990 to 4.45 per 100,000 in 2021 (Lin et al., 2024). By 2019, an estimated 4.9–5.0 million people worldwide were living with IBD, with crude prevalence rising from about 3.3 million in 1990 to 4.9 million in 2019 (Park et al., 2023; Wang et al., 2023). The pathogenesis of IBD involves multiple interlinked components, including immune dysregulation, genetic susceptibility, epithelial barrier dysfunction, and environmental triggers (Calvez et al., 2025). Over the past decade, there has been increasing recognition that chronic intestinal inflammation in IBD has become increasingly evident, and it arises not only from persistent immune cell activation but also from profound disruptions in epithelial cell homeostasis, barrier integrity, and cellular stress response pathways (Calvez et al., 2025; Guan, 2019). Among these, endoplasmic reticulum (ER) stress and the unfolded protein response (UPR) have emerged as central regulatory mechanisms linking the pathological onset of intestinal inflammation and mucosal damage (Zheng et al., 2025; Ma et al., 2017). ER stress refers to a cellular condition in which the folding capacity of the ER is disrupted, leading to the accumulation of misfolded or unfolded proteins within the ER lumen (Chen et al., 2023). The ER is essential for protein folding, calcium homeostasis, and lipid metabolism; however, under inflammatory, oxidative, or metabolic disregulation, ER stress is triggered, leading to activation of the UPR (English and Voeltz, 2013; Singh et al., 2024). UPR is an adaptive cellular mechanism that helps to restore ER homeostasis by suppressing protein synthesis, optimizing protein folding, and eliminating misfolded proteins (Hetz et al., 2020). However, persistent ER stress can trigger apoptosis, autophagy, and modulation of inflammatory pathways, leading to epithelial barrier disruption, immune system activation, and sustained intestinal inflammation (Chen et al., 2023). Moreover, genetic polymorphisms in ER stress-related genes such as X-box-binding protein 1 (*XBP1*) and Orosomucoid-like protein 3 (*ORMDL3*) have been associated with increased IBD susceptibility (Vanhove et al., 2018; Kaser et al., 2008). In intestinal epithelial cells (IECs) and immune cells, persistent ER stress promotes inflammation by enhancing pro-inflammatory cytokine release, impairing mucosal healing, and altering microbiota composition (Cao, 2018). In addition to intracellular stress mechanisms, extracellular signaling pathways have gained attention for their role in regulating immune and epithelial responses. Among these, extracellular vesicles (EVs), particularly exosomes, are increasingly recognized as key mediators of intercellular communication in intestinal homeostasis and inflammation (Wu et al., 2024). Exosomes are small membrane-bound vesicles (30–150 nm) that carry proteins, lipids, and nucleic acids, and play major roles in immune regulation, maintaining epithelial barrier integrity, modulating gut microbial communities, and facilitating intercellular communication in the intestinal microenvironment (Chavda et al., 2023; Kalluri and LeBleu, 2020; Ayyar and Moss, 2021). It has been reported that ER stress is mechanistically linked to exosome biogenesis, influencing secretory dynamics and altering the molecular content of exosomes, thereby modulating their biological activity (Ye and Liu, 2022; Jahangiri et al., 2022). ER stress-induced exosomes may carry specific microRNAs or proteins that promote inflammatory responses or enhance tumor cell survival and dissemination. For instance, immune cell-derived exosomal miR-155 and miR-21 contribute to inflammation by targeting regulatory pathways essential for maintaining immune balance (Nail et al., 2023). Furthermore, exosomes derived from ER-stressed cells may influence immune responses by modulating the activity of macrophages, dendritic cells (DCs), and T cells, thereby worsening the inflammatory landscape within the intestinal microenvironment (Zhang et al., 2019). The dynamic relationship between ER stress and exosomes may play a significant role in the development of IBD. Elucidating how ER stress influences the composition and function of exosomes, and how these vesicles, in turn, affect intestinal homeostasis and immune responses, could provide novel insights into the underlying mechanisms of IBD. In this review, we aim to discuss the current research exploring how ER stress influences exosome biogenesis, alters their molecular composition, and modulates their biological functions within the intestinal microenvironment. This review further examines how ER stress–induced exosomes contribute to intestinal inflammation, epithelial barrier dysfunction, and immune dysregulation, thereby promoting IBD progression. In addition, the review also highlights the potential of targeting ER stress–exosome interactions as a future therapeutic approach for the management of IBD.

## 2 Endoplasmic reticulum (ER) stress

**FIGURE 1:**
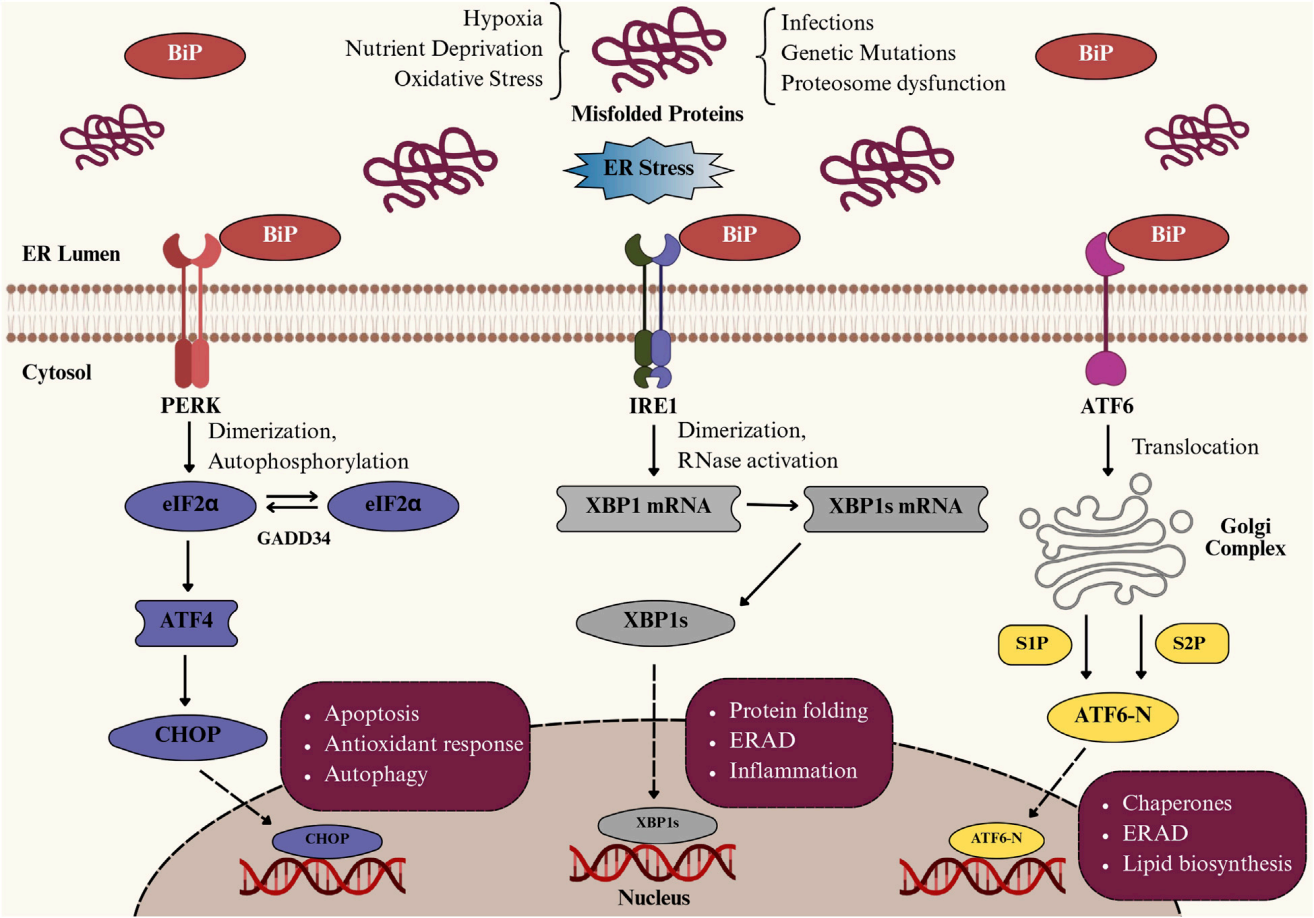
Pathways of the unfolded protein response (UPR) activated during endoplasmic reticulum (ER) stress. Schematic representation of unfolded protein response (UPR) activation in intestinal epithelial cells under ER stress. Accumulation of misfolded proteins due to hypoxia, oxidative stress, or nutrient deprivation activates three key sensors: PERK, IRE1, and ATF6. PERK phosphorylates eIF2α, inducing ATF4/CHOP-mediated apoptosis and autophagy; IRE1 splices XBP1 mRNA, generating XBP1s that regulate protein folding, ER-associated degradation (ERAD), and inflammation; and ATF6 translocates to the Golgi, where cleavage produces ATF6-N, which upregulates chaperones and lipid biosynthesis to restore ER homeostasis.

The ER is a multifunctional organelle composed of interconnected tubules and cisternae that extend throughout the cytoplasm, playing a central role in protein synthesis, protein folding, post-translational modifications, lipid metabolism, and calcium homeostasis (Singh et al., 2024; Deka et al., 2022; Girigoswami et al., 2023). Under physiological conditions, chaperone proteins such as Glucose-Regulated Protein 78 (GRP78)/BiP and protein folding enzymes including calnexin and calreticulin maintain protein quality control; however, factors like oxidative stress, hypoxia, nutrient deprivation, calcium imbalance, genetic mutations, and inflammation can disrupt ER function, causing the accumulation of misfolded or unfolded proteins and thereby inducing ER stress (Table 1) (Chen et al., 2023; Singh et al., 2024). This activates the UPR, an adaptive mechanism regulated by three primary ER membrane sensors, including protein kinase RNA-like ER kinase (PERK), inositol-requiring enzyme 1 (IRE1), and activating transcription factor-6 (ATF6) (Chen et al., 2023), as illustrated in Figure 1. Upon ER stress, GRP78 dissociates, enabling PERK to oligomerize and phosphorylate eIF2α, which transiently inhibits overall protein translation while selectively enhancing Activating Transcription Factor 4 (ATF4) translation. ATF4 promotes expression of genes involved in amino acid metabolism, antioxidant defense, and ER-associated degradation (ERAD), but also triggers C/EBP homologous protein (CHOP), a transcription factor that suppresses Bcl-2, upregulates pro-apoptotic mediators (BIM, PUMA), and promotes reactive oxygen species (ROS) generation and calcium release, thereby initiating mitochondrial stress (Verjan Garcia et al., 2023; Hu et al., 2019; Smolková et al., 2020). IRE1, once activated by oligomerization and autophosphorylation, splices XBP1 mRNA to generate the transcriptionally active XBP1s, which enhance the transcription of genes related to ERAD, protein folding, and lipid synthesis (Park et al., 2021; Jiang et al., 2015). Prolonged IRE1 activation also leads to Regulated IRE1-Dependent Decay (RIDD), c-Jun N-terminal Kinase (JNK) activation *via* TRAF2–ASK1, and degradation of specific microRNAs, thereby linking ER stress to inflammation and apoptosis (Siwecka et al., 2021; Hotamisligil, 2010). Meanwhile, upon release from GRP78, ATF6 translocates to the Golgi, where it is sequentially cleaved by site-1 and site-2 proteases (S1P and S2P). This cleavage generates the cytosolic fragment ATF6p50, which migrates to the nucleus and activates genes encoding ER chaperones (GRP78, GRP94), protein-folding enzymes (PDIs), and components of ERAD (Hetz et al., 2020; Yamamoto et al., 2007). Persistent ER stress overcomes these pathways, leading to apoptosis, inflammation, and cellular dysfunction, particularly in highly secretory cells like IECs (Salminen et al., 2020; Song et al., 2008; Lin et al., 2008).

**TABLE 1:** Cellular factors contributing to ER stress and their associated pathological outcomes.

Factor	Mechanism	Pathological impact	References
Oxidative stress	Disrupts redox balance, inhibits disulfide bonds.	Protein misfolding, inflammation, cancer, IBD.	Cao and Kaufman (2014)
Calcium disruption	Impairs chaperone activation (calnexin, calreticulin).	ER dysfunction, IBD, and neurodegeneration.	Stutzmann and Mattson (2011); Mekahli et al. (2011)
Nutrient deprivation	Affects the folding and glycosylation of the proteins.	UPR activation, tumor stress response.	Chen and Shoulders (2024)
Hypoxia	Reduces ATP and the folding capacity of protein.	Tumor survival, metastasis.	Akman et al. (2021)
Infections	Overload of the ER by viral/bacterial proteins.	Inflammation, IBD	Da Fonseca et al. (2024)
Genetic mutations	Causes persistent misfolded proteins.	Cystic fibrosis, IBD, ER overload.	Chen et al. (2023)
Inflammatory cytokines	Disrupt signalling pathways; induce ER stress.	Chronic inflammation, IBD, autoimmunity.	Salminen et al. (2020)

### 2.1 Intracellular and extracellular matrix–mediated stress in IBD

IBD arises from a complex interaction between intracellular stress responses and extracellular matrix (ECM)-mediated stress, both of which play pivotal roles in sustaining chronic intestinal inflammation, epithelial barrier disruption, and fibrosis (Zhang et al., 2025; Lin M. et al., 2025). At the cellular level, ER stress disrupts epithelial cell function by activating the UPR (He et al., 2025). When this adaptive mechanism fails, it triggers apoptosis, compromises barrier integrity, and promotes pro-inflammatory signaling cascades that sustain mucosal inflammation (Kong et al., 2024; Otte et al., 2023). These intracellular changes disrupt cellular communication and tissue regeneration, fostering a microenvironment that promotes chronic inflammation (Saez et al., 2023). Concurrently, extracellular stress arises from ECM remodeling, where the ECM undergoes significant compositional and mechanical alterations (Prakash and Shaked, 2024). Normally, the ECM provides a structural and regulatory framework for the cells, but during IBD progression, excessive deposition of collagen and fibronectin, fragmentation of hyaluronan, and upregulation of matrix metalloproteinases (MMPs) disrupt ECM homeostasis (Zhao et al., 2025; Petrey and de la Motte, 2017). These changes increase tissue stiffness and alter cell–matrix interactions, promoting fibroblast activation, angiogenesis, and immune cell infiltration (Petrey and de la Motte, 2017; Lin S. N. et al., 2025). The remodeled ECM imposes additional mechanical and biochemical stress on epithelial and mesenchymal cells *via* altered mechanotransduction pathways, thereby sustaining inflammation and promoting fibrogenesis (Mierke, 2024; Horta et al., 2023). Mechanical cues at the cellular level further influence ER stress development. Cells constantly experience forces from tissue tension, fluid shear, and ECM stiffness, which are transmitted through integrins and cytoskeletal networks to intracellular organelles (Mierke, 2024). Elevated mechanical load, such as that found in regions of stiffened ECM or fibrotic tissue, can deform the ER membrane, disrupt protein folding, and enhance ER stress signaling (Lenna and Trojanowska, 2012). Likewise, changes in osmotic pressure or cytoskeletal tension can disrupt ER-calcium homeostasis, which further amplifies the UPR (Carreras-Sureda et al., 2018). The cytoskeleton also acts as a critical mechanotransductive bridge between ECM remodeling and intracellular ER stress (Huang et al., 2023). Comprised of actin filaments, microtubules, and intermediate filaments, the cytoskeleton transmits mechanical and biochemical signals from the ECM to intracellular organelles, including the ER (Momotyuk et al., 2025; Gurel et al., 2014). Alterations in ECM stiffness, composition, or ligand availability are detected by integrins and focal adhesion complexes, which transmit these mechanical cues through the cytoskeleton to intracellular organelles, particularly the ER, thereby modulating its structure, calcium homeostasis, and protein-folding capacity (Di et al., 2023; Derricks et al., 2015; Khoonkari et al., 2024). Conversely, ER stress can modify cytoskeletal composition through UPR-mediated signaling, specifically affecting actin filament dynamics and microtubule stability, which, in turn, influence cell shape, motility, and mechanotransduction (van Vliet and Agostinis, 2017; Khoonkari et al., 2024). This coordinated interplay may establish a dynamic network in which ECM remodeling, cytoskeletal dynamics, and ER stress mutually amplify each other, driving epithelial barrier disruption, immune activation, and fibrotic remodeling in IBD.

### 2.2 ER stress–mediated immune dysregulation and molecular outcomes in intestinal epithelium

**FIGURE 2:**
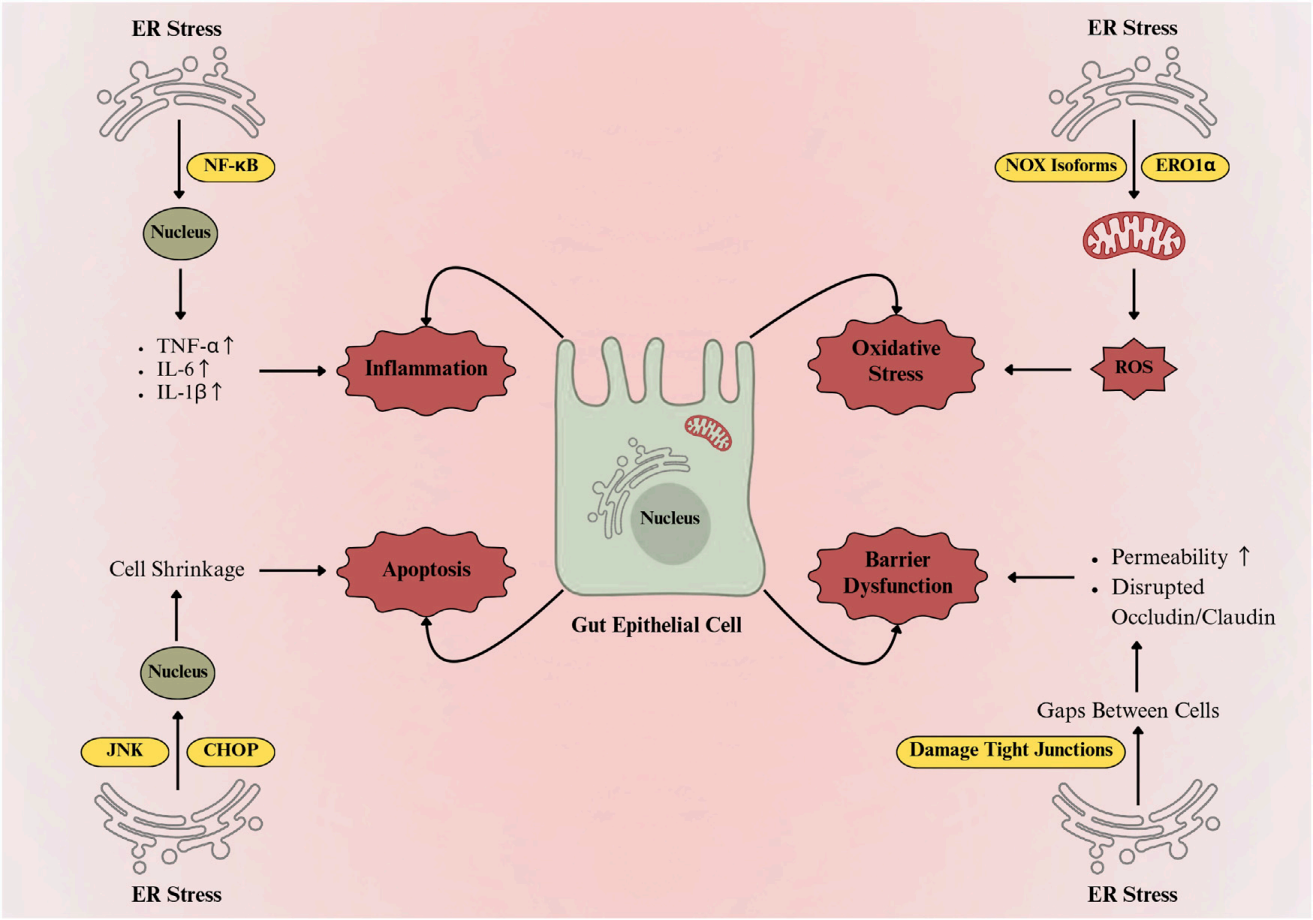
ER stress-mediated damage and functional impairment in gut epithelial cells. ER stress activates NF-κB and JNK/CHOP pathways, inducing proinflammatory cytokines (TNF-α, IL-6, IL-1β) and apoptosis. Concurrently, ROS generation *via* NADPH oxidase isoforms and ER oxidoreductin-1α disrupts tight junctions, increases epithelial permeability, and impairs intestinal barrier integrity.

Gut homeostasis depends on a balanced immune–epithelial interface that regulates tolerance and inflammation (Bretto et al., 2025). Paneth and goblet cells, derived from Lgr5^+^ intestinal stem cells, are central to mucosal defence (Leushacke and Barker, 2014). Paneth cells sense microbes *via* MyD88-dependent Toll-like receptors and secrete antimicrobial peptides RegIII-β, RegIII-γ, and α-defensins (Yang et al., 2025), while goblet cells produce Mucin 2 (MUC2) to form a protective mucus barrier. Defective MUC2 folding or glycosylation compromises barrier function and predisposes to colitis and colorectal cancer (Kang et al., 2022). Different physiological and pathological conditions can produce ER stress, triggering the UPR. However, if ER stress persists, UPR imbalance can drive programmed cell death and inflammatory responses, which are particularly critical in intestinal epithelial cells that rely on UPR for their normal function (Di Mattia et al., 2025). Patients with active IBD often exhibit increased ER stress markers in both ileal and colonic epithelial tissues (Rodrigues et al., 2025; Cao, 2018). IBD patients also show signs of impaired integrated stress response, including decreased phosphorylation of eukaryotic initiation factor 2 alpha (eIF2α), which normally protects against oxidative and ER stress, infection, and inflammation. The IRE1α-XBP1 signaling pathway plays a critical role in intestinal immune modulation. Knockdown of XBP1 in IECs promotes ER stress, triggers inflammatory responses, and induces Paneth cell apoptosis (Luo and Cao, 2015). Prolonged ER stress activates all three UPR branches (IRE1α, PERK, and ATF6), leading to inflammation and apoptosis (Martinotti and Ranzato, 2025). IRE1α–TRAF2 activates JNK (Riaz et al., 2020), while PERK–ATF4–CHOP signalling induces apoptosis and NF-κB–driven cytokine transcription (Rozpedek et al., 2016). ATF6 supports chaperone synthesis but also amplifies CHOP and NF-κB expression (Chen et al., 2023). Sustained UPR activation disrupts tight junction proteins and epithelial permeability (Di Mattia et al., 2025). Prolonged ER stress also induces oxidative stress, mitochondrial dysfunction, and lipid peroxidation, resulting in excessive reactive oxygen species (ROS) production that damages lipids, proteins, and DNA, impairs mitochondrial function, and disrupts tight junction proteins such as occludin, claudins, and ZO-1, thereby compromising epithelial integrity and increasing intestinal epithelial permeability (Loncke et al., 2021; Bhandary et al., 2012). In gut epithelium, prolonged ER stress also triggers a highly regulated adaptive response known as autophagy, which acts as a protective mechanism to maintain cellular homeostasis (Shi et al., 2024). However, mutations in the autophagy-related genes *ATG16L1*, *IRGM*, and *NOD2* linked to Crohn’s disease disrupt autophagy and thereby intensify ER stress (Alula and Theiss, 2023). These effects are closely linked to the major molecular and cellular consequences of prolonged ER stress in IECs, as illustrated in Figure 2. Recent studies (Ye and Liu, 2022; Jahangiri et al., 2022) indicate that ER stress not only drives inflammation, apoptosis, autophagy, and oxidative stress but also affects cell-to-cell communication *via* exosomes. ER stress influences exosome biogenesis and cargo composition, which in turn contributes to intestinal inflammation (Zhou et al., 2025). The role of ER stress in modulating exosome-mediated communication in IBD will be discussed in detail below.

## 3 Exosomes in intestinal inflammation

### 3.1 Overview of exosomes and their molecular cargo

Exosomes are nano-sized (30–150 nm) EVs of endosomal origin, secreted by almost all cell types, and play key roles in intercellular communication. They carry a broad spectrum of bioactive molecules, including proteins, lipids, metabolites, mRNA, DNA, and non-coding RNAs such as miRNAs, lncRNAs, and circRNAs (Kumar et al., 2024; Ruivo et al., 2017; Banerjee et al., 2020). In IBD, exosomes contribute to chronic inflammation by promoting immune cell activation, epithelial barrier disruption, and pro-inflammatory signaling (Ocansey et al., 2020). Exosomal proteins such as tetraspanins (CD9, CD63), ALIX, TSG101, and heat shock proteins (Hsp70, Hsp90) are associated with the amplification of intestinal inflammation and stress-induced epithelial damage (Schopf et al., 2017; Mazurov et al., 2013). miRNAs like miR-21, miR-126, and miR-23a, frequently enriched in exosomes, amplify inflammatory responses, promote angiogenesis, and compromise barrier integrity in the gut mucosa (Grimolizzi et al., 2017; Hu et al., 2020). Similarly, exosomal lncRNAs, including H19 and UCA1, are associated with promoting epithelial-mesenchymal transition and influencing immune regulatory pathways that intensify mucosal inflammation and contribute to therapeutic resistance (Xia et al., 2023; Fan et al., 2014). Also, exosomal circRNAs, such as circ-IARS and circ-DLEU2, affect immune signaling and epithelial function by binding to specific miRNAs and blocking their normal gene regulatory activities (Li et al., 2018; Wu et al., 2018).

### 3.2 Exosome biogenesis in IBD

**FIGURE 3:**
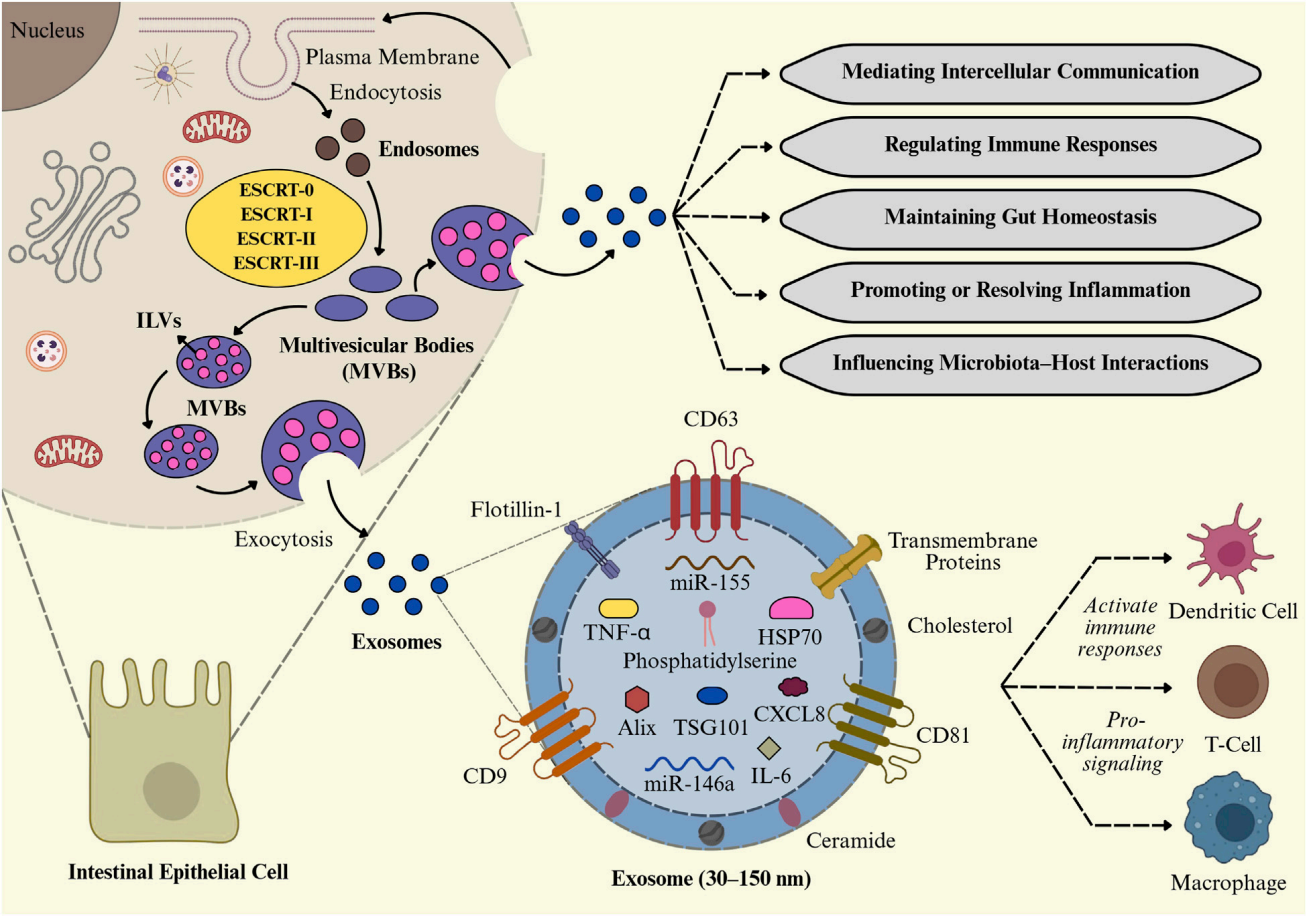
Biogenesis and functional roles of intestinal exosomes. Exosome formation in intestinal epithelial cells (IECs) *via* the ESCRT-mediated multivesicular body (MVB) pathway. These exosomes, enriched with cytokines (TNF-α, IL-6), miRNAs (miR-155, miR-146a), and proteins (CD63, TSG101, HSP70), regulate intercellular communication, immune responses, gut homeostasis, and microbiota–host interactions, while also contributing to proinflammatory signaling and immune cell activation.

The endosomal system generates tiny EVs, as part of the intricate intracellular process known as exosome biogenesis, as illustrated in Figure 3 (Gurung et al., 2021). It begins with the inward budding of the plasma membrane to form early-sorting endosomes, which can integrate cargo from both the extracellular environment and intracellular compartments such as the Golgi apparatus and ER (Xie et al., 2022). As early-sorting endosomes mature into late-sorting endosomes, they undergo a second inward budding process, forming multivesicular bodies (MVBs) that include numerous intraluminal vesicles (ILVs), which are exosome precursors (Kalluri and LeBleu, 2020). Exosome biogenesis involves not only membrane budding and vesicle formation but also highly regulated molecular machinery that decides which proteins, lipids, and nucleic acids are packaged into ILVs (Ha et al., 2016). This sorting process is mainly carried out by a set of protein complexes collectively known as the endosomal sorting complex required for transport ESCRT), as well as by ESCRT-independent mechanisms. The ESCRT pathway comprises four main protein complexes, including ESCRT-0, I, II, and III, that work together to regulate vesicle fission, membrane budding, and the recognition of ubiquitinated cargo (Ha et al., 2016; Zhang et al., 2019). ESCRT-0 first recognizes, and confines ubiquitinated cargo proteins on the endosomal membrane, followed by the recruitment of ESCRT-I and ESCRT-II to assist membrane deformation. ESCRT-III completes vesicle fragmentation with ATPase VPS4 (Dai et al., 2020). Proteins like TSG101 and ALIX facilitate cargo selection and membrane budding (Taha et al., 2019). In the ESCRT-independent mechanism, ceramides derived from sphingomyelin promote vesicle budding through membrane curvature and phase separation (Horbay et al., 2022). Tetraspanins such as CD9, CD63, and CD81 organize membrane microdomains and assist in cargo sorting *via* tetraspanin-enriched microdomains (Toribio and Yáñez-Mó, 2022). MVBs may either fuse with lysosomes for cargo degradation or with the plasma membrane for extracellular exosome release. This secretion is mediated by Rab GTPases (Rab27a/b, Rab11), SNARE proteins, and syntenin-1, which regulate membrane trafficking and fusion (Xu et al., 2022; Kalluri and LeBleu, 2020). Released exosomes are 30–150 nm in diameter and appear cup-shaped with the transmission electron microscope or spheroidal under electron microscopy (Jung and Mun, 2018; Fakhredini et al., 2022). It has been reported that exosomes released by immune cells such as DCs, macrophages, T cells, and B cells actively contribute to the pathogenesis of IBD by persistent inflammation, modulating immune responses, and altering intestinal homeostasis (Hazrati et al., 2022). The role of these exosomes derived from the immune cells is discussed below.

### 3.3 Role of immune cell-derived exosomes in IBD

The immune microenvironment of the intestinal mucosa is significantly influenced by the immune cell-derived exosomes, which provide effective communication *via* both autocrine and paracrine pathways. By carrying proteins, lipids, and nucleic acids, immune cell-derived exosomes can alter the function of other immune cells, either promoting or inhibiting immunological responses (Hazrati et al., 2022). Depending on the cytokine environment, macrophages can polarize into pro-inflammatory M1 or anti-inflammatory M2 phenotypes (Shapouri-Moghaddam et al., 2018). Pro-inflammatory mediators such as TNF-α, IL-6, IL-23, and miR-155 are prevalent in exosomes released by M1 macrophages. These exosomes may increase inflammation by stimulating NLRP3 inflammasomes, Toll-like receptors, and TNF-related pathways in recipient cells (Wang et al., 2019; Tian et al., 2022). Additionally, these exosomes promote the migration of leukocytes and sustained inflammatory signaling in the intestinal mucosa, which are characteristics of IBD, by increasing the expression of endothelial cell adhesion molecules like ICAM-1 (Osada-Oka et al., 2017). Similarly, exosomes secreted by T cells, including CD8^+^ cytotoxic T cells, CD4^+^ helper T cells, and regulatory T cells (Tregs), serve as carriers of bioactive molecules, including proteins, lipids, and microRNAs, which influence target cell behavior (Table 2). Exosomes derived from activated T cells activate the RAS/MAPK signaling cascade, inducing ERK phosphorylation and amplifying inflammatory responses in recipient immune cells, thereby contributing to IBD pathophysiology (Zhang et al., 2024). Exosomes produced by CD8^+^ T cells, which include cytotoxic substances, such as granzyme and perforin, could contribute to chronic inflammation by causing tissue damage. Exosomes generated from CD4^+^ T cells interact with immune cells, including macrophages and NK cells, and may alter immunological activities by stimulating TCRs to release major histocompatibility complex (MHC) class II-like peptides (Wang and Shi, 2022). Upon activation of B cells *via* B cell receptor or Toll-like receptor (TLR) signaling, B cells release exosomes rich in immune molecules, including MHC I/II, costimulatory markers (CD40, CD80, CD86), and B cell marker CD19. These exosomes can stimulate T cell responses by presenting antigens through MHC-II-peptide complexes (Azoulay-Alfaguter and Mor, 2018). However, this activation frequently results in a proliferation of T cells with limited capacity to proliferate and a decreased production of cytokines (Hazrati et al., 2022), which may be a contributing factor to the persistent inflammation observed in IBD. Likewise, DCs produce exosomes that vary in function depending on their maturation status and the local cytokine environment. DC progenitors develop into mature DCs under pro-inflammatory conditions, which are characterized by high levels of cytokines like IL-6 and TNFα (Hazrati et al., 2022). These cells then produce exosomes that are enriched with immunostimulatory molecules such as MHC class II, CD40, CD80, and CD86 (Tkach et al., 2017). Naïve T cells are extensively activated by these mature DC-derived exosomes, leading them to differentiate into effector T cells and increasing the adaptive immune response. Moreover, these exosomes promote the proliferation and activation of natural killer cells by expressing IL-15 receptor alpha (IL-15Rα) and NKG2D ligands such as MIC-A, MIC-B, and ULBP-1, resulting in increased IFN-γ secretion that impairs mucosal inflammation (Bassani et al., 2019; Viaud et al., 2009). DC-derived exosomes also increase cytokine production and immune activation by releasing inflammatory microRNAs, such as miR-155, to recipient immune cells. Their integrin and adhesion molecule content facilitates the influx of immune cells to inflamed tissues, contributing to the longevity of inflammation (Lindenbergh et al., 2019). Thus, mature DC-derived exosomes in IBD function as potent pro-inflammatory agents that drive immune cell activation, promote the production of cytokines, and maintain mucosal dysregulation, thereby worsening the severity and progression of IBD.

**TABLE 2:** Key immune cell-derived exosomes and their functional roles in IBD.

Immune cell type	Key exosomal cargo	Effect in IBD	Inflammatory role	References
Macrophages (M1/M2)	M1: TNF-α, IL-6, IL-23, miR-155 M2: TGF-β, IL-10, miR-21-5p, miR-155-5p	M1 promotes leukocyte recruitment and inflammation; M2 reduces inflammation but may promote tumorigenesis	Pro-inflammatory (M1)/Anti-inflammatory, pro-tumorigenic (M2)	Wang et al. (2019); Tian et al. (2022); Ma et al. (2021)
T Cells (CD4+/CD8+)	Granzyme, perforin, MHC II-like peptides	Cytotoxicity, cytokine release, and immune activation	Pro-inflammatory	Wang and Shi, (2022); Zhang et al. (2024)
Dendritic cells	MHC II, CD40, CD80, CD 86, IL-15Rα, MIC-A/B, ULBP-1, miR-155	Stimulate T/NK cells; mucosal inflammation	Strongly pro-inflammatory	Tkach et al. (2017); Bassani et al. (2019)
Neutrophils	Myeloperoxidase, miR-23a, miR-155	Barrier damage, impaired wound healing	Strongly pro-inflammatory	Ayyar and Moss (2021)
Mast cells	Histamine, IL-4, TNF-α, tryptase, chymase, miR-223	Increase permeability, promote fibrosis	Pro-inflammatory	Li et al. (2020)
Natural killer cells	Perforin, granzyme B, IFN-γ, miR-186	Epithelial injury and inflammation	Pro-inflammatory	Hazrati et al. (2022)

## 4 Exosome-mediated miRNA dysregulation and barrier dysfunction in IBD

In IBD, exosomes carrying dysregulated miRNAs and inflammatory cargo contribute to gut barrier breakdown, microbial imbalance, and inflammation. Exosomal miR-223 derived from immune cells downregulates Claudin-8 and CLDN8 expression, leading to the disruption of tight junctions and increased epithelial permeability through activation of the IL-23 pathway. This, in turn, promotes microbial translocation and enhances inflammatory cell infiltration (Li et al., 2020; Zhou et al., 2021). Similarly, elevated miR-21 and miR-301a in epithelial exosomes, induced by pro-inflammatory stress, promote *AKT* activation and suppress cadherin-1 or *PTEN* expression, disrupting junctional integrity and promoting inflammation-driven permeability (Zhou et al., 2021). Moreover, aberrant exosomal miR-29a expression in IECs has been linked to reduced occludin levels and enhanced epithelial permeability, particularly in aging and inflammatory conditions marked by elevated levels of IFN-γ, IL-6, and IL-1β (Park et al., 2022). These exosomes can also promote macrophage polarization toward the pro-inflammatory M1 phenotype through delivery of miR-155, which enhances NF-κB activity and the secretion of TNFα and IL-6, amplifying epithelial injury and mucosal immune activation (Shen et al., 2021). Moreover, exosomes derived from the serum of IBD patients have been found to upregulate Interleukin-8 (IL-8) in epithelial cells, triggering macrophage chemotaxis and sustaining immune cell infiltration, which further disrupts the mucosal barrier and disturbs microbial homeostasis (Shen et al., 2021). Furthermore, exosomal cargo altered by infection with *Escherichia coli* strains exhibiting adherent-invasive characteristics, such as the downregulation of let-7b, can alter macrophage activation, hinder autophagy, and activate fibrogenic and ER stress pathways, thereby intensifying CD progression (Carrière et al., 2016). Notably, host-derived exosomes can also directly influence microbial gene expression. Fecal exosomes containing miR-515-5p or miR-1226-5p can enter gut bacteria like *Fusobacterium nucleatum* or *E. coli*, altering their transcriptional activity, favoring the expansion of pro-inflammatory species, and contributing to dysbiosis (Liu et al., 2016). Additionally, disruptions in Rab27a-mediated exosomal secretion from myeloid cells can limit the release of anti-inflammatory miRNAs such as miR-146a, leading to excessive macrophage activation and worsening colitis severity (Bauer et al., 2022). In addition, *F. nucleatum-*derived EVs have been shown to intensify the experimental colitis by disrupting epithelial barrier function and promoting inflammatory autophagy (Wei S. et al., 2023). In Dextran Sulfate Sodium (DSS)-treated mice, oral administration of *F. nucleatum-*derived EVs significantly worsened colitis severity by downregulating miR-574-5p and activating the CARD3-dependent autophagy pathway in IECs, which led to reduced expression of tight junction proteins ZO-1 and occludin, elevated IL-1β, IL-6, and TNFα secretion, and pronounced epithelial damage (Wei Z. et al., 2023). Wei S. et al. (2023) employed a DSS-induced colitis mouse model along with an experiment on cultured IECs to investigate the effects of *F. nucleatum*-derived EVs. The study utilized targeted knockdown and overexpression approaches to dissect the role of miR-574-5p and the CARD3-dependent autophagy pathway and quantified tight junction proteins and pro-inflammatory cytokines using standard molecular and immunological techniques. Inhibition of autophagy or targeting the miR-574-5p/CARD3 axis alleviated both barrier disruption and colitis symptoms, highlighting a direct pathogenic mechanism by which bacterial EVs can impair gut integrity in IBD (Wei Z. et al., 2023).

## 5 Interconnection of ER stress and exosome biogenesis in IBD

The interconnection between ER stress and exosome release may significantly contribute to the pathogenesis and progression of IBD, with significant implications for intestinal inflammation and immune modulation. Inherited genetic variations in major ER stress regulators such as XBP1, ARG2, and ORMDL3, along with external factors like microbial-derived molecules and pro-inflammatory cytokines, can significantly impair ER homeostasis (Kaser and Blumberg, 2010). This dysregulation of ER stress can activate pro-inflammatory signaling pathways in the gut, reduce the integrity of the mucosal barrier, and induce epithelial cell apoptosis (Qiao et al., 2021). ER stress, arising from the accumulation of misfolded proteins, activates the UPR, leading to alterations in cellular homeostasis, and this stress response not only influences intracellular functions but also modulates EV dynamics, particularly exosome biogenesis (Ye and Leu, 2022). ER stress influences the formation of MVBs and the release of exosomes, thereby establishing a crucial link between intracellular stress responses and intercellular communication (Jahangiri et al., 2022). As exosomes serve as crucial mediators of intercellular communication, in the context of IBD, their interplay with ER stress may play an important role in disease onset, progression, and recovery. Under ER stress conditions, cells initiate adaptive mechanisms to restore homeostasis, which not only regulate protein folding and degradation but also influence cellular processes such as autophagy and exosome formation (Senft and Ronai, 2015; Jahangiri et al., 2022). It has been reported that ER stress enhances the formation of MVBs, the precursors to exosomes, by promoting the inward budding of late endosomes (Wu et al., 2021). This process is facilitated by the upregulation of proteins involved in vesicle trafficking and membrane remodeling, such as CD63 (Gurunathan et al., 2021). Consequently, ER stress may lead to an increase in exosome secretion, thereby altering the extracellular matrix and influencing neighboring cells. Exosomes derived from ER-stressed cells often carry stress-related biomolecules, including proteins such as GRP78 and CHOP, lipids such as ceramides and lysophosphatidylcholine, and RNAs, including miR-23a, which can transmit ER stress and activate inflammatory signals in recipient cells (Ye and Leu, 2022). In IBD, where the intestinal epithelium is subjected to chronic inflammatory stimuli, this mechanism amplifies persistent ER stress, disrupting epithelial cell function and integrity and contributing to barrier dysfunction and increased intestinal permeability (Shi et al., 2024). Simultaneously, enhanced exosome production under ER stress conditions may facilitate the transfer of pro-inflammatory mediators, including cytokines, microRNAs, and damaged proteins, to adjacent cells (Ye and Leu, 2022). In the mucosal microenvironment of IBD, enriched with inflammatory mediators and damage-associated molecular patterns (DAMPs), this ER stress–exosome interplay creates a feedback loop that continues and amplifies inflammation (Ocansey et al., 2020). Exosomes derived from stressed epithelial cells can deliver inflammatory signals to immune cells, continuing the inflammatory characteristic of IBD, while the altered protein and RNA composition of exosomes under ER stress may influence the differentiation, activation, and polarization of immune cells such as macrophages, dendritic cells, and T cells, thereby worsening the inflammatory response (Ocansey et al., 2020). The mutual interaction between ER stress and exosome biogenesis may further intensify the pathophysiology of IBD, as exosomes not only serve as vehicles for transmitting stress-induced signals (Liao et al., 2019) but also participate in the modulation of ER stress pathways in recipient cells (Kanemoto et al., 2016). This intercellular communication loop may underscore the complexity of cellular adaptations to stress and highlights the potential for exosomes to propagate pathological conditions beyond their cell of origin. A more detailed analysis of how ER stress interfaces with exosome biogenesis in IBD will be discussed in the following sections.

### 5.1 ER stress-induced exosome release and cargo modifications

Under ER stress, cells upregulate pathways that significantly enhance exosome biogenesis and secretion. Kanemoto et al. (2016) demonstrated that ER stress significantly enhances multivesicular body formation and exosome secretion, establishing a direct link between ER stress signaling and extracellular vesicle biogenesis using HeLa cells as well as IRE1α/β and PERK knockout mouse embryonic fibroblasts (MEFs), treated with tunicamycin. By inhibiting the key UPR sensors, the stress-related increase in exosome release was prevented. This is because certain key molecules involved in the generation of exosomes, such as ESCRT proteins and ceramide, are activated by ER stress pathways, particularly those involving IRE1α and PERK (Ye and Leu, 2022). This highlights a strong connection between ER stress and increased exosome production. Likewise, triggering intense ER stress in choriocarcinoma cells with tunicamycin results in a significant increase in the release of EVs (Collet et al., 2018). These findings suggest that increased exosome production is a common cellular response to stress, indicating that cells deliberately adjust exosome release to cope with stressful conditions (Collet et al., 2018). ER stress also changes the composition of exosomal cargo. Under stress, cells are more likely to load vesicles with DAMPs and proinflammatory molecules. In a study by Collet et al. (2018), it was revealed that inducing severe ER stress in placental cells led to the release of EVs that were enriched with high levels of DAMPs, HMGB1, and HSP70. These EVs containing DAMPs naturally promote inflammation. In the metabolic context, obesity-induced metabolic stress, which is characterized by elevated palmitate levels, activates NF-κB and ER stress pathways in adipocytes, which in turn significantly enhance the enrichment of certain microRNAs into exosomes (Li et al., 2024). Similarly, inflammatory signals in the gut, such as cytokines and hypoxia, are likely to alter the RNA composition of exosomes. In fact, exosomal miRNA profiles are disrupted in IBD; for instance, miR-21, a pro-inflammatory miRNA elevated in inflamed colonic tissue, is selectively incorporated into epithelial cell-derived exosomes in response to stress-related stimuli like substance P/NK-1R activation (Bakirtzi et al., 2019). Similarly, neutrophil-derived exosomes carry miRNAs like miR-23a and miR-155, which negatively affect colonic epithelial wound healing (Ayyar and Moss, 2021). Additionally, exosomal miR-223 from macrophages has been shown to intensify intestinal barrier damage in a DSS-induced colitis model (Chang et al., 2023). Also, IBD patient-derived exosomes contain a highly pro-inflammatory molecular profile. Mitsuhashi et al. (2016) collected intestinal luminal aspirates (∼10 mL of fluid) from the colon of IBD patients and healthy controls during colonoscopy to isolate luminal EVs and compare their molecular contents. It has been reported that EVs from IBD patients contained significantly elevated levels of proinflammatory cytokines IL-6, IL-8, TNFα, and the anti-inflammatory cytokine IL-10, along with specific proteins and miRNAs associated with inflammation. It also demonstrated that these EVs could induce proinflammatory responses in cultured IECs and macrophages, suggesting that they originate from ER-stressed intestinal epithelial and immune cells within inflamed mucosal tissue and actively contribute to disease-associated inflammation.

### 5.2 Exosome-mediated transfer of ER stress signals in intestinal inflammation

Exosomes released from ER-stressed IECs can transmit stress signals to neighboring cells within the gut microenvironment. Gut-resident secretory cells under proteostatic stress, such as Paneth or goblet cells with XBP1 dysfunction, are particularly prone to releasing exosomes enriched with UPR components or misfolded proteins (Bhattacharya and Chatterji, 2024). Mahadevan et al. (2011) revealed that macrophages exposed to conditioned medium from ER-stressed tumor cells were used to investigate intercellular communication of stress signals. They observed that macrophages not only upregulate UPR genes but also produce elevated levels of proinflammatory cytokines, demonstrating that ER stress can be transmitted from tumor cells to recipient myeloid cells, thereby activating the UPR and promoting an inflammatory response Moreover, ER stress-associated exosomes have the potential to modulate immune responses. He et al. (2020) found that macrophages exposed to exosomes derived from ER-stressed HepG2 cells significantly increase the expression of IL-6, MCP-1, and IL-10. These exosomes might contain phospholipids or vesicular miRNAs that activate NFκB or JNK signaling pathways, or they may carry UPR-related transcription factors or chaperones like GRP78 either within their cargo or on their surface, thereby amplifying stress responses in recipient cells. Functionally, recipient cells react by triggering UPR sensors, elevating cytokine production, and frequently exhibiting impaired barrier integrity. For instance, exosomal miR-21 can target tight junction regulators and promote epithelial migration, while other miRNAs or proteins in IBD EVs may disrupt mucosal integrity (Bakirtzi et al., 2019). Furthermore, neutrophil-derived exosomes, which are abundant in inflamed tissues, secrete myeloperoxidase and specific miRNAs (e.g., miR-23a and miR-155). These miRNAs are taken up by IECs and have been shown to impair wound healing (Ayyar and Moss, 2021). Collectively, these mechanisms suggest that ER stress in a single cell can be transmitted through exosomes, triggering ER stress markers along with inflammatory responses in nearby gut cells. A summary of the exosome-mediated interactions and their functional consequences in IBD is represented in Table 3.

**TABLE 3:** Exosome-mediated propagation of ER stress and inflammatory signals in IBD.

Source of exosomes	Key cargo	Effect on intestinal microenvironment	References
ER-stressed IECs derived exosomes	dsDNA-rich exosomes	Activate cGAS–STING in macrophages → excessive cytokine release	Zhou et al. (2021)
IBD patient-derived exosomes	Pro-inflammatory cytokines, miRNAs, proteins	Increased IL-8 production, disrupted barrier integrity	Mitsuhashi et al. (2016); Bakirtzi et al. (2019)
Neutrophil-derived exosomes	miR-23a, miR-155, myeloperoxidase	Impaired wound healing, barrier damage	Ayyar and Moss (2021)
Adipose tissue exosomes	miR-155	Promote M1 polarization, worsen colitis	Wei Z. et al. (2023)
Salivary exosomes	Multiple inflammatory mediators	Disrupt tight junctions, worsen colitis	Yang et al. (2024)

### 5.3 Exosome-ER stress loop in gut inflammation

**FIGURE 4:**
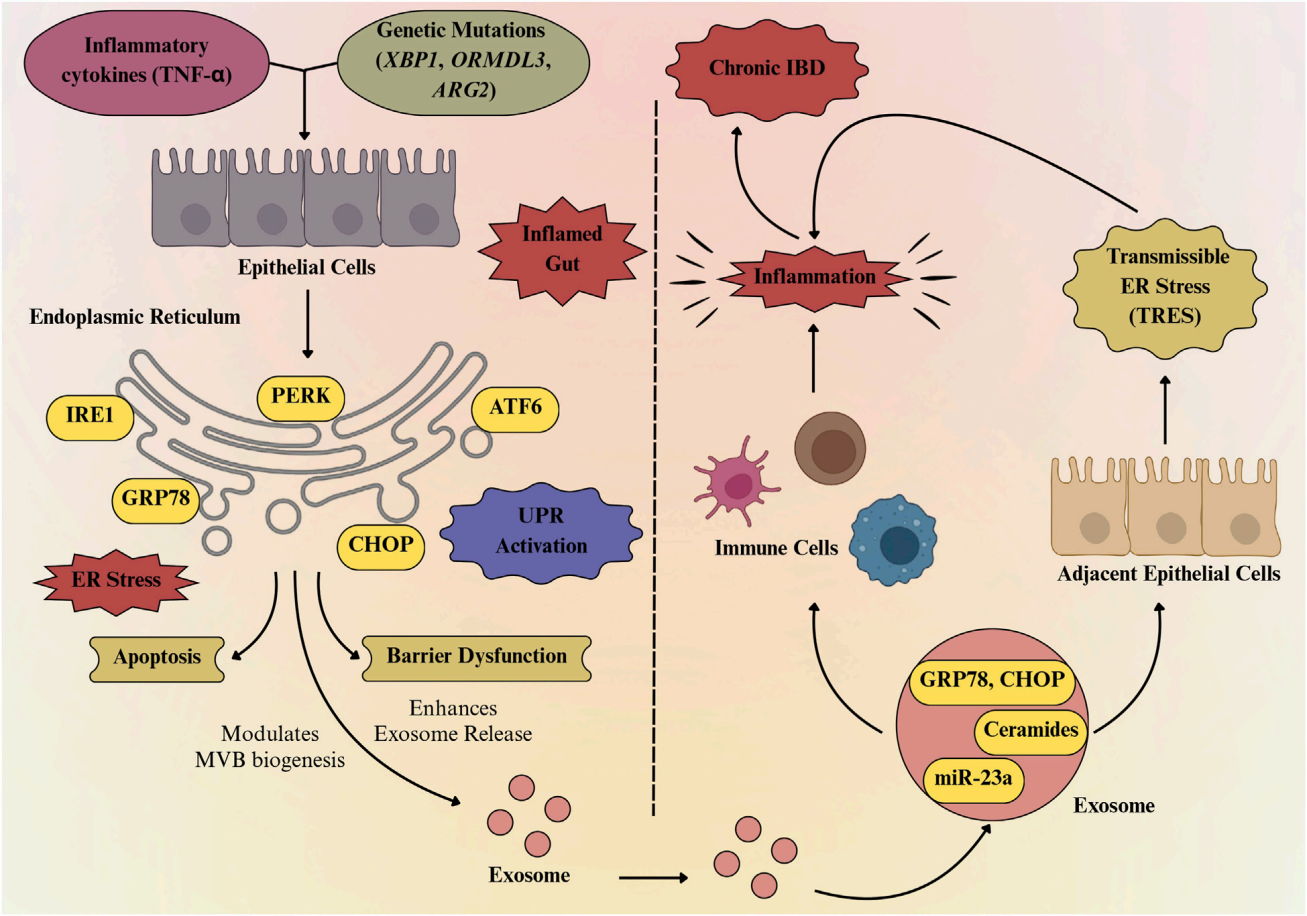
Exosome-mediated bidirectional loop linking ER stress to chronic gut inflammation. Inflammatory cytokines and genetic mutations (XBP1, ORMDL3, ARG2) trigger ER stress in intestinal epithelial cells, activating the unfolded protein response (UPR) through PERK, IRE1, and ATF6 pathways. Persistent ER stress induces CHOP-mediated apoptosis and barrier dysfunction, while also promoting exosome release enriched with GRP78, CHOP, ceramides, and miR-23a. These exosomes propagate inflammation, activate immune cells, and transmit ER stress to neighboring cells, thereby sustaining chronic IBD.

Exosomes carrying inflammatory and ER stress signals amplify cytokine activity and cellular dysfunction, which subsequently enhances ER stress and triggers further exosome release (Figure 4). For instance, Mitsuhashi et al. (2016) showed that exosomes from IBD patients promote macrophage migration and cytokine release, while IL-8-containing exosomes attract neutrophils, collectively exacerbating ER stress in epithelial cells. Notably, this ER stress significantly influences the molecular composition of the exosomes, leading to their enrichment with specific proteins such as Pregnancy zone protein (Shao et al., 2021) as well as lipids like ceramides associated with IRE1α activation (Dasgupta et al., 2020), and potentially distinct miRNAs (miR-223, miR-23a, and miR-155) that reflect the inflammatory and stressed state of the originating cell (Wang et al., 2020; Ayyar and Moss, 2021). At the molecular level, exosomes carrying miRNAs or DAMPs can strongly stimulate the NF-κB and JAK/STAT signaling pathways in immune cells. Mahadevan et al. (2011) demonstrated that macrophages exposed to tumor-derived ER stress signals not only activate their own stress response pathways but also acquire a pronounced proinflammatory phenotype. These macrophages showed increased levels of stress-related markers such as Grp78, Gadd34, CHOP, and spliced Xbp1, indicating that they were deeply affected by the tumor environment. Also, Chen et al. (2022) showed that a high-fat diet worsens TNBS-induced colitis in mice *via* mesenteric adipose tissue-derived exosomes enriched with *MALAT1*. These exosomes are taken up by colonic epithelial cells, where *MALAT1* suppresses miR-15a-5p, leading to activation of the ATF6-mediated ER stress pathway. This cascade results in amplified inflammatory signaling, increased epithelial cell damage, and worsening of colitis pathology, highlighting the interplay between diet, exosome signaling, and ER stress in intestinal inflammation. Similarly, Jin et al. (2025) demonstrated that IEC-specific Prdx3 deficiency leads to severe DSS-induced colitis through increased oxidative stress and exosomal release of miR-1260b. These exosomes impair epithelial barrier integrity and activate p38 MAPK/NF-κB signaling, promoting inflammation. This further supports that the stress-induced exosomal cargo amplifies inflammatory signaling and epithelial dysfunction, thereby sustaining ER stress and contributing to disease severity in IBD.

## 6 Preclinical evidence linking ER stress–exosome to IBD pathogenesis

Growing experimental evidence reveals that ER stress in IECs promotes exosome-mediated immune activation, thereby amplifying intestinal inflammation. Zhou et al. (2021) demonstrated that ER-stressed IECs, in both DSS-induced colitis models and in colonic biopsies from CD patients, secrete exosomes enriched with mitochondrial and nuclear double-stranded DNA (dsDNA), suggesting a mechanism by which ER stress contributes to intestinal inflammation. When intestinal macrophages absorb these exosomal dsDNAs, they function as strong DAMPs that trigger the cytosolic cGAS–STING DNA-sensing pathway. This activation leads to the induction of proinflammatory cytokines, including TNF-α, IL-6, and IFN-β (Zhou et al., 2021). Additionally, in DSS-induced colitis mice, therapeutic suppression of exosome secretion with GW4869 not only decreased STING activation in immune cells but also reduced the severity of the disease, suggesting a transmissible loop between immunological activation and epithelial stress. Furthermore, Kaser et al. (2008) showed that deletion of XBP1 in IECs leads to spontaneous enteritis and increased vulnerability to colitis as a result of Paneth cell malfunction and epithelial hyperactivity to inflammatory and microbial stimuli such as TNFα and flagellin. The study revealed and validated the link between XBP1 genetic variants and both forms of human IBD (UC and CD), with novel hypomorphic variants emerging as risk factors. By activating macrophages and disrupting the intestinal barrier, Gong et al. (2022) showed that serum-derived exosomes from individuals with CD increase intestinal inflammation. These exosomes increased macrophage count and proinflammatory cytokine expression *in vitro*, while also enhancing epithelial permeability. The study also demonstrated how CD altered the exosomal miRNA profile, specifically lowering the levels of let-7b-5p (Gong et al., 2022). The let-7b-5p/TLR4 pathway was found to be a major mechanistic regulator of inflammation mediated by macrophages. These findings collectively highlight a pathogenic feedback loop in which ER stress in the gut epithelium induces the release of pro-inflammatory exosomes, which in turn activate immune responses that further intensify ER stress and epithelial injury (Gong et al., 2022). This emerging axis provides novel insights into the chronicity of intestinal inflammation in IBD and identifies potential therapeutic targets such as UPR modulators and exosome release inhibitors to disrupt the pathological interplay between epithelial stress responses and immune activation. Also, Yang et al. (2024) demonstrated that salivary exosomes from active IBD patients aggravated colitis in mice. These exosomes disrupted intestinal epithelial integrity by reducing tight junction proteins in Caco-2 cells and induced inflammatory responses in THP-1 macrophages, potentially intensifying ER stress in IECs. Moreover, the distinct miRNA profiles found in these salivary exosomes indicate that their cargo may influence cellular stress pathways, immune responses, and gut microbiota balance (Yang et al., 2024). Another study by Wei S. et al. (2023) showed that visceral adipose tissue-derived exosomes from high-fat diet-fed mice intensified DSS-induced colitis by shifting their miRNA cargo toward a pro-inflammatory profile, particularly enriching miR-155. These exosomes infiltrated the intestinal lamina propria and promoted M1 macrophage polarization, thereby enhancing intestinal inflammation. Key preclinical evidences connecting ER stress and exosomes in IBD pathogenesis is summarized in (Table 4).

**TABLE 4:** Preclinical insights into ER stress–exosome–mediated intestinal inflammation.

Source/Model	Key findings	Type of study	References
DSS-induced colitis mice	ER-stressed IECs release dsDNA-rich exosomes → activate cGAS–STING in macrophages → excessive cytokine release.	*In vivo / ex vivo*	Zhou et al. (2021)
XBP1-deficient mice	XBP1 deletion → spontaneous enteritis, Paneth cell dysfunction, ↑ colitis susceptibility.	*In vivo*	Kaser et al. (2008)
Serum exosomes (from CD patients)	↑ macrophage cytokines, ↑ epithelial permeability; ↓ let-7b-5p/TLR4 drives inflammation.	*In vitro / ex vivo*	Gong et al. (2022)
Salivary exosomes (from IBD patients)	Disrupted tight junctions in Caco-2 cells induced macrophage inflammation, worsened colitis.	*In vitro / in vivo*	Yang et al. (2024)
Adipose exosomes (from high-fat diet mice)	miR-155-enriched exosomes → M1 macrophage polarization → worsened DSS colitis.	*In vivo*	Wei S. et al. (2023)

## 7 Therapeutic and diagnostic implications

**FIGURE 5:**
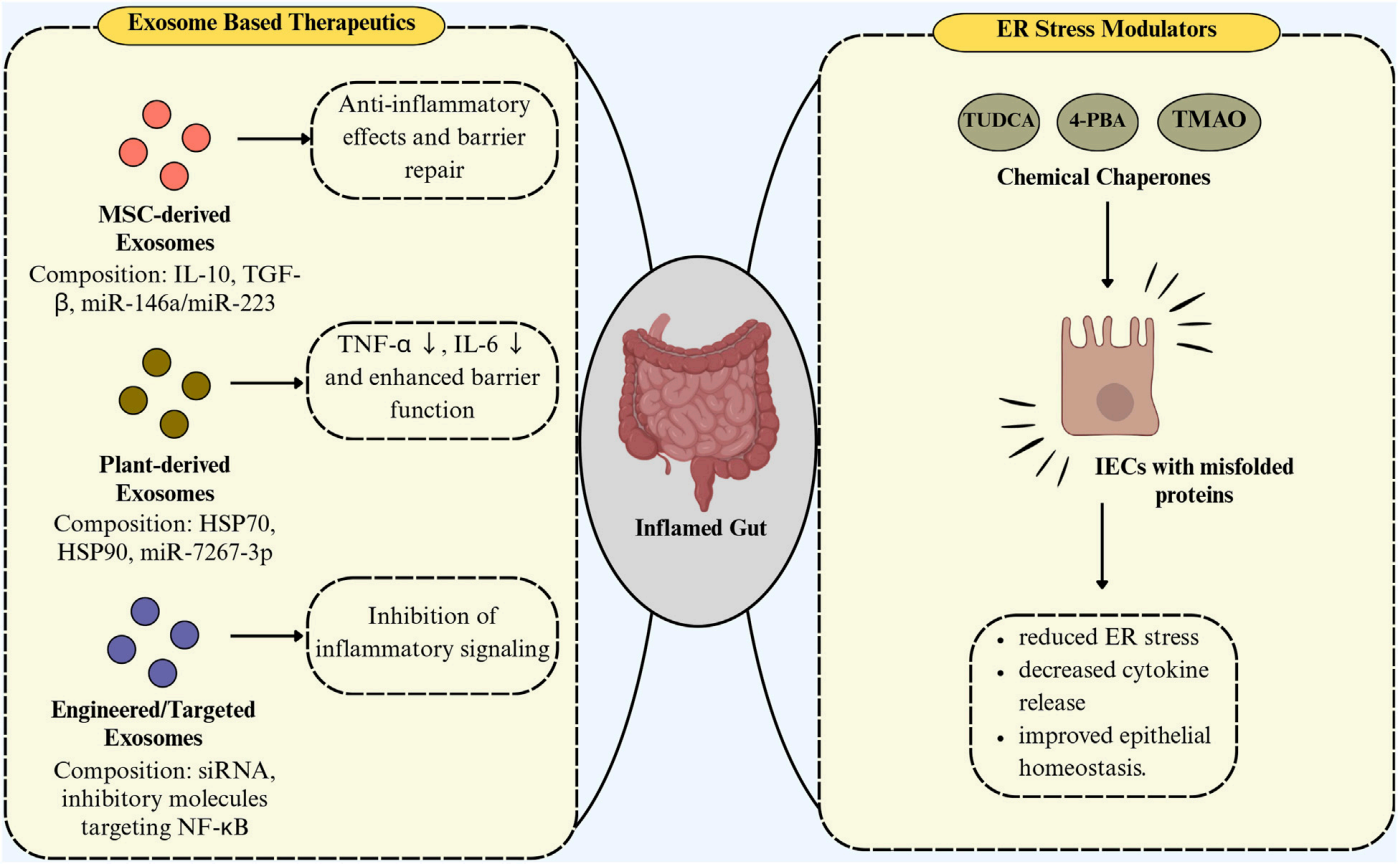
Exosome-based therapeutics and ER stress modulators in intestinal inflammation. Therapeutic strategies for intestinal inflammation include exosome-based approaches—MSC-derived exosomes promoting anti-inflammatory effects and barrier repair, plant-derived exosomes reducing TNF-α/IL-6 and enhancing barrier function, and engineered exosomes inhibiting NF-κB signaling—and ER stress modulators (TUDCA, 4-PBA, TMAO) acting as chemical chaperones to alleviate ER stress, decrease cytokine release, and restore epithelial homeostasis.

Exosomes play a dual role in the pathogenesis and treatment of IBD, acting both as mediators of inflammation and as potential therapeutic agents. Exosomes display a context-dependent balance between pro-inflammatory and anti-inflammatory functions. For instance, serum-derived exosomes enriched with proinflammatory mediators, such as let-7b-5p, can activate macrophages *via* the p38/ERK pathway, thereby intensifying colitis and promoting tissue damage (Huang et al., 2025). In contrast, mesenchymal stem cell–derived exosomes (e.g., from adipose or umbilical cord sources) are enriched with anti-inflammatory cargos, particularly those carrying IL-10, TGF-β, and miR-146a. These exosomes suppress NF-κB signaling, reduce cytokine production, and promote epithelial repair, highlighting their therapeutic promise (Lee et al., 2023; Kheradmand et al., 2025). A study by Banerjee et al. (2015) has demonstrated that UCMSCs effectively prevented DSS-induced colitis in immunodeficient mice by reducing inflammation, tissue damage, and ER stress, highlighting the therapeutic potential of targeting ER stress in IBD. The study also showed that UCMSC-derived factors can likely directly modulate intestinal injury, suggesting a novel diagnostic and treatment approach for IBD. Recent studies (Wei Z. et al., 2023; Saleem et al., 2024) highlight the ability of exosomes to carry bioactive molecules that influence immune responses and epithelial integrity, making them desirable targets for therapeutic intervention. Various sources of exosomes have been explored as a therapeutic target, including those derived from mesenchymal stem cells, immune cells, intestinal epithelial cells, and even plant and food-derived nanovesicles (Ocansey et al., 2020). Several emerging therapeutic strategies targeting exosomes from different sources and ER stress modulation have shown promise in alleviating IBD. Exosomes derived from mesenchymal stem cells, particularly those isolated from adipose tissue or bone marrow, have demonstrated anti-inflammatory and immunomodulatory properties that reduce intestinal inflammation and promote mucosal healing in colitis models (Wei S. et al., 2023; Zubair et al., 2025). These exosomes can carry beneficial microRNAs like miR-146a, miR-223, which inhibit NF-κB activation and suppress pro-inflammatory cytokine expression in macrophages and IECs, thereby restoring immune balance (Zubair et al., 2025). Additionally, plant-derived exosome-like nanoparticles from ginger, grape, or broccoli are gaining attention due to their natural origin and ability to deliver bioactive molecules directly to the gut (Li et al., 2023). It has been reported that, in DSS-induced colitis models, ginger-derived nanoparticles were found to be predominantly absorbed by intestinal epithelial and immune cells, decreasing pro-inflammatory cytokines (TNF-α, IL-6), and improving barrier function (Zhang et al., 2016). Another study by Deng et al. (2017) has revealed that broccoli-derived nanoparticles (BDNs) play a protective role in mouse models of colitis by modulating DC function through activation of AMP-activated protein kinase (AMPK). BDNs induced the formation of tolerogenic DCs and suppressed their activation, thereby maintaining intestinal immune homeostasis (Deng et al., 2017). The anti-inflammatory activity was primarily associated with the sulforaphane component of BDNs, since defense against DSS-induced colitis was exclusively provided by AMPK^+/+^ DCs and BDN lipids, including sulforaphane (Deng et al., 2017). Moreover, in mouse models of IBD, it has been demonstrated that chemical chaperones like tauroursodeoxycholic acid and 4-phenylbutyric acid reduce ER stress by stabilizing protein conformation and reducing the accumulation of unfolded proteins, which restores epithelial homeostasis and decreases inflammation (Cao et al., 2013). Thus, a dual approach including modulating the origin and cargo of exosomes, and thereby reducing ER stress, may offer a combinational potential in decreasing IBD severity and promoting gut homeostasis. A schematic representation of these therapeutic opportunities is illustrated in Figure 5.

### 7.1 Human clinical trials

Exosome research is gradually advancing toward clinical application, with several early-phase human trials underway (https://clinicaltrials.gov/). While most of the exosome-based studies focus on cancer, regenerative medicine, and drug delivery, an increasing number of studies are now exploring the potential roles of exosomes in IBD. A Phase I clinical trial (NCT06853522) is currently investigating the safety and therapeutic potential of human umbilical cord mesenchymal stem cell–derived exosomes (hUC-MSC-Exos) in patients with active UC. The trial seeks to determine whether hUC-MSC-Exos can be safely administered to patients while also providing early indications of their ability to alleviate intestinal inflammation and promote mucosal healing. In addition, a randomized pilot study (NCT04879810) is evaluating the safety and therapeutic potential of ginger-derived exosomes, with or without curcumin, in patients with IBD. This trial assesses whether exosomes alone or in combination with curcumin can reduce symptoms, improve disease scores, and modulate inflammatory biomarkers. The study plans to enroll up to 90 patients across three groups, with the combination therapy expected to provide greater symptom improvement, supporting the development of novel nutraceutical-based approaches for patients with persistent IBD. Similarly, a Phase I clinical trial (NCT06742203) is evaluating WPMDE1, a whey protein milk-derived exosome nutritional supplement, in healthy adults. The trial aims to assess tolerability, usability, and optimal dosing of WPMDE1, with participants receiving ascending doses over 7–30 days. Blood sampling, vital sign monitoring, and gastrointestinal symptom tracking will be performed to guide the design of future studies targeting patients with irritable bowel syndrome and IBD. Likewise, phase I/II clinical studies (NCT05499156, NCT05402748) are evaluating human placenta–derived MSC exosomes for the treatment of complex perianal or anal fistulas in CD patients who have not responded to prior therapies. In both studies, exosomes were injected directly into the fistula tract weekly for three consecutive weeks, and patients were monitored for fistula healing by MRI, changes in inflammatory biomarkers (CRP, IL-6, TNF-α, calprotectin), and quality of life over 12 weeks. These studies provide preliminary evidence that MSC-derived exosomes may promote fistula closure, reduce local inflammation, and improve patient-reported outcomes, supporting their potential as a novel therapeutic option in Crohn’s-related perianal disease. A summary of these human clinical trials, highlighting their design, interventions, and key findings in IBD, is provided in Table 5.

**TABLE 5:** Human clinical trials of exosome-based therapies in IBD.

Trial ID	Sources of exosomes	Phase of study	Intervention/Design	Key outcomes
NCT06853522	hUC-MSC	Phase I	Administration of hUC-MSC-Exosomes in UC patients	Evaluation of safety, inflammation reduction, and mucosal healing
NCT04879810	Ginger with/without curcumin	Pilot study	Exosomes with/without curcumin supplementation	Assessment of symptoms, disease activity scores, and biomarkers
NCT06742203	Whey protein milk-derived	Phase I	Ascending dose administration over 7–30 days	Determination of tolerability, optimal dosing, and gastrointestinal symptom response
NCT05499156	Placenta-derived MSC	Phase I/II	Weekly injections of exosomes into the fistula tract	Evaluation of fistula healing, biomarker modulation, and quality of life improvement
NCT05402748	Placenta-derived MSC	Phase I/II	Weekly injections of exosomes into the fistula tract	Evaluation of fistula closure and inflammation.

## 8 Challenges and future directions

Despite significant advances in understanding the ER stress–exosome axis in IBD, several key challenges and substantial knowledge gaps remain, particularly concerning how these processes operate in human disease and how current insights can be effectively translated into clinical applications. Most of the recent research is based on animal models or *in vitro* cell cultures, which do not accurately represent the complexity of the human gut, leading to variability and inconsistencies in results across studies. This complicates the understanding of the interaction between exosomes and ER stress in IBD patients. Moreover, few clinical studies directly validated the role of ER stress–exosome interactions in IBD, leaving a critical gap between preclinical findings and patient outcomes. Another major challenge is the lack of advanced models that can accurately mimic the complex environment of the human intestine. Conflicting results regarding exosome cargo composition, release mechanisms, and functional effects further underscore the need for more robust, standardized investigations. Current experimental models are limited in replicating the intricate microenvironment of the human gut. Although emerging tools such as organoids (mini-gut models) (Yin et al., 2019), gut-on-a-chip systems (Ashammakhi et al., 2020), and single-cell technologies (Zheng, 2023) offer promising platforms, yet they remain in the early stages of development and are not fully explored. Technical limitations in isolating, characterizing, and analyzing exosomes, due to their heterogeneity and overlap with other EVs, (Tzng et al., 2023), hinder reproducibility, and limit the discovery of reliable biomarkers or therapeutic targets. From the clinical application point of view, ensuring the safety, specificity, and scalability of exosome-based therapies, as well as addressing complex regulatory requirements, are additional challenges. (Rezaie et al., 2022). Future research should focus on elucidating the precise molecular mechanisms by which ER stress pathways, such as PERK, IRE1α, and ATF6, regulate exosomal cargo loading, including proteins, lipids, and miRNAs that influence intestinal inflammation. To better understand the dynamic connection between ER stress and exosomes during disease progression and treatment, and to identify novel diagnostic and therapeutic approaches for IBD, future studies could employ a range of experimental strategies. CRISPR/Cas9-mediated gene editing could be utilized to selectively modulate ER stress components (PERK, IRE1α, or ATF6) or miRNA cargo regulators to determine their specific roles in exosomal cargo loading and intestinal inflammation (Xu et al., 2025). Engineered exosomes carrying therapeutic miRNAs or silencing RNAs could be developed and tested in organoid models to assess their capacity to ameliorate ER stress-induced inflammation. Integration of spatial transcriptomics like GeoMx or 10x Xenium platforms on intestinal biopsies from IBD patients will enable precise mapping of the cellular microenvironments where ER stress and exosome interactions occur within inflamed tissue, revealing the spatial relationship between stressed epithelial cells and immune infiltrates (Liu et al., 2024; Mennillo et al., 2024). Single-cell and spatial transcriptomic profiling of patient-derived intestinal organoids will bridge the gap between *in vitro* mechanistic studies and clinical pathophysiology (Tsalikis et al., 2016). These combined approaches will provide unprecedented resolution of how exosomal cargo heterogeneity impacts localized inflammatory microenvironments. Co-immunoprecipitation coupled to mass spectrometry (Co-IP/MS) can be used to identify protein-protein interactions within exosome cargo complexes and to monitor changes in these interactions in response to ER stress induction (Fu et al., 2024). RNA immunoprecipitation (RNA-IP) followed by high-throughput sequencing should be employed to identify ER stress-responsive miRNAs and their interaction with RNA-binding proteins within exosomes, elucidating selective cargo loading mechanisms (Wozniak et al., 2020). Machine learning algorithms could also be applied to multi-omics datasets like proteomics, lipidomics, and transcriptomics to identify predictive biomarker profiles within exosomes that distinguish disease states and predict treatment response (Lin M. et al., 2025). Deep learning models trained on exosomal cargo profiles could facilitate the development of advanced diagnostic tools for early IBD detection and patient stratification. Additionally, live-cell imaging to track exosome biogenesis and trafficking under stress conditions, and longitudinal human studies to validate findings and monitor disease progression. Collectively, these complementary methodologies could provide a comprehensive framework for understanding ER stress-exosome interactions in IBD.

## 9 Conclusion

ER stress and exosomes are recognized as two critical contributors to IBD pathogenesis. Persistent ER stress not only disrupts epithelial integrity and immune homeostasis but also alters exosome biogenesis and cargo composition, enabling the transmission of stress and inflammatory signals within the intestinal microenvironment. Previous experimental studies have demonstrated that ER stress can modulate exosome composition, including miRNAs, proteins, and lipids, which in turn affect immune responses and barrier function in gut epithelium, highlighting a mechanistic link between ER stress and exosome-mediated signaling. ER stress in IECs has shown altered exosomal miRNA profiles, such as increased miR-21 and miR-155, which target tight junction proteins and inflammatory regulators, thereby intensifying epithelial permeability and cytokine production. Conversely, stress-induced exosomes have been shown in *in vitro* and *in vivo* models to amplify immune dysregulation and epithelial dysfunction. ER stress–induced exosomes derived from IECs, or macrophages have been shown to activate NF-κB and the NLRP3 inflammasome in murine models of colitis, supporting their role in amplifying mucosal inflammation. Additionally, inhibition of the IRE1α–XBP1 pathway or blocking exosome release using GW4869 has been shown to mitigate colitis severity, providing functional validation of this mechanism. Despite these advances, the molecular profiles of ER stress–derived exosomes and their precise contributions to intercellular communication remain incompletely defined, and further experimental validation is needed to establish their clinical relevance as biomarkers or therapeutic targets. Overall, the current evidences suggest that the dynamic connection between ER stress and exosome-mediated signaling is a key mediator of chronic inflammation and epithelial dysfunction in IBD, providing novel insights into disease mechanisms and potential therapeutic strategies aimed at restoring gut homeostasis. Further mechanistic studies, together with advances in exosome engineering and ER stress–targeted interventions, could offer innovative approaches for improving clinical outcomes in IBD.

## Glossary

AMPK: AMP-Activated Protein Kinase
ATF4: Activating Transcription Factor 4
ATF6: Activating Transcription Factor 6
BDNs: Broccoli-Derived Nanoparticles
CD: Crohn’s Disease
CHOP: C/EBP Homologous Protein
DAMPs: Damage-Associated Molecular Patterns
DCs: Dendritic Cells
DNA: Deoxyribonucleic Acid
dsDNA: Double-Stranded DNA
DSS: Dextran Sulfate Sodium
ER: Endoplasmic Reticulum
ERAD: ER-Associated Degradation
EVs: Extracellular Vesicles
eIF2α: Eukaryotic Initiation Factor 2 alpha
ESCRT: Endosomal Sorting Complex Required for Transport
GRP78: Glucose-Regulated Protein 78
HSP: Heat Shock Protein
IL-1β: Interleukin-1 beta
IL-6: Interleukin-6
IL-8: Interleukin-8
IECs: Intestinal Epithelial Cells
IRE1: Inositol-Requiring Enzyme 1
MAPK: Mitogen-Activated Protein Kinase
MHC: Major Histocompatibility Complex
MSC: Mesenchymal Stem Cells
MUC2: Mucin 2
NF-κB: Nuclear Factor kappa B
ORMDL3: Orosomucoid-like protein 3
PERK: Protein kinase RNA-like Endoplasmic Reticulum Kinase
ROS: Reactive Oxygen Species
TNFα: Tumor Necrosis Factor-alpha
TLR: Toll-Like Receptor
UPR: Unfolded Protein Response
XBP1: X-box Binding Protein 1
